# Ubp2 Regulates Rsp5 Ubiquitination Activity *In Vivo* and *In Vitro*

**DOI:** 10.1371/journal.pone.0075372

**Published:** 2013-09-19

**Authors:** Mandy H. Y. Lam, Andrew Emili

**Affiliations:** Banting and Best Department of Medical Research, Donnelly Centre for Cellular and Biomolecular Research, Department of Molecular Genetics, University of Toronto, Toronto, Ontario, Canada; University of Minnesota, United States of America

## Abstract

The yeast HECT-family E3 ubiquitin ligase Rsp5 has been implicated in diverse cell functions. Previously, we and others [1], [2] reported the physical and functional interaction of Rsp5 with the deubiquitinating enzyme Ubp2, and the ubiquitin associated (UBA) domain-containing cofactor Rup1. To investigate the mechanism and significance of the Rsp5-Rup1-Ubp2 complex, we examined Rsp5 ubiquitination status in the presence or absence of these cofactors. We found that, similar to its mammalian homologues, Rsp5 is auto-ubiquitinated *in vivo*. Association with a substrate or Rup1 increased Rsp5 self-ubiquitination, whereas Ubp2 efficiently deubiquitinates Rsp5 *in vivo* and *in vitro.* The data reported here imply an auto-modulatory mechanism of Rsp5 regulation common to other E3 ligases.

## Introduction

Rsp5, the sole member of the Nedd4 E3 ligase family in *S. cerevisiae*, has been implicated in various steps in intracellular trafficking, including endocytosis of plasma membrane proteins [3] such as the Fur4 uracil permease [4] and the Mat α receptor Ste2 [5]. Apart from a key initial role in substrate ubiquitination immediately preceding target internalization, Rsp5 is involved in other steps in vesicle trafficking, such as sorting at the multivesicular body (MVB) [6], [7], [8] that precedes cargo delivery and degradation at the vacuole, and more recently, in mediating Golgi to ER trafficking [9].

Similar to other Nedd4 ligases, the Rsp5 protein contains specific domains which are important for function: an N-terminal C2 lipid binding domain, important for subcellular localization to endosomal membranes [10], three WW protein binding domains in the central region important for the recruitment of substrates and binding to accessory cofactors, and a Homologous to the E6-AP Carboxyl Terminus (HECT) catalytic domain on the C-terminal end of the protein, responsible for ligase activity [11].

Regulation of Rsp5 activity is known to occur by various mechanisms, including binding to various adaptor proteins through specific protein-protein interactions. For example, the Rsp5 cofactors Bul1 and Bul2 have been implicated in dual roles in the Rsp5-mediated sorting of the amino acid permease Gap1 by facilitating ubiquitination at the plasma membrane [12], and by dictating trafficking of newly synthesized Gap1 from the Golgi to the vacuole by specifying poly-ubiquitin chain length, presumably through modulation of Rsp5 activity [13]. In addition, recognition of substrates by Rsp5 can occur either by direct binding through its WW domains, or indirectly through additional adaptors [14], [15], [16], [17], [18], [19], [20].

An additional potential route of ligase regulation may occur through E3 ubiquitination, either through an intra-molecular (*cis*) or an inter-molecular (*trans*) mechanism. In almost all cases studied to date, E3 auto-ubiquitination results in the down-regulation of ligase activity through proteasome-associated degradation, as in the case of the mammalian ligase Nedd4-2 [21], responsible for sorting of transmembrane proteins in a manner analogous to yeast Rsp5. However, non-proteolytic ubiquitination has also been reported, such as for the mammalian Itch ligase [22] involved in immune signaling [23], [24].

Unlike most other E3 ubiquitin ligases, the ubiquitination of Rsp5 has not been well characterized, and a catalytically inactive mutant is comparably stable as wildtype Rsp5 [25]. While Rsp5 has been reported in large scale studies to be ubiquitinated [26], [27], [28], [29] and to become auto-ubiquitinated [30], [31], [32], the physiological significance of this modification has not been demonstrated.

E3 ligases can also be regulated through the intra-molecular folding of the enzyme. This has been reported for several mammalian cases, including Itch and Nedd4-2 [21]. In the case of Nedd4-2, ligase activity is catalytically inhibited in the absence of substrate by physical associations between its different domains [21]; the WW domains on Nedd4-2 binds to a PY motif located within the HECT catalytic domain of the protein. Whether this occurs through a *cis* or *trans* (i.e. intra- vs inter-molecular) mechanism is not yet known. Nevertheless, this binding inactivates the enzyme. Presumably, unfolding and release occurs upon the recognition and interaction with a substrate, resulting in ‘on demand’ re-activation [21], leading to the rapid ubiquitination and down-regulation of both the substrate and the ligase itself by 26S proteasome degradation.

Association with a deubiquitinating enzyme (DUB) is another increasingly recognized mode of E3 regulation. Many E3 ligases, for example the mammalian RING ligase Mdm2 [33], as well as Itch [34], are normally complexed with DUBs *in vivo*. In general, deubiquitinating enzymes have been reported to cleave off ubiquitin from auto-ubiquitinated ligases, resulting in protein stabilization.

Previous studies in yeast by our group and others [1], [2] have shown that Rsp5 interacts both physically and functionally with the ubiquitin specific protease Ubp2. Ubp2 was reported to be important for proper trafficking at the MVB of Rsp5 membrane protein substrates, including the uracil permease Fur4 [2]. The interaction of Ubp2 and Rsp5 is stabilized by the co-factor Rup1, which binds to both Ubp2 and Rsp5 but has an unclear physiological role [1], [2]. Although the exact role of Ubp2 and Rup1, is uncertain, they have been implicated as positive effectors of Rsp5-dependent ubiquitination [2], [35].

Given the accumulating evidence from studies showing auto-ubiquitination as an important mode of E3 regulation in mammalian systems, we examined if Rsp5 is auto-ubiquitinated under physiological circumstances in yeast and whether Ubp2 modulates this enzyme state. Here, we show for the first time that a significant fraction of Rsp5 is extensively poly- and auto-ubiquitinated *in vivo*, but this form of Rsp5 is both resistant to proteolysis and normally rapidly reversed by Ubp2. This effect is direct since Ubp2 is able to efficiently deubiquitinate recombinant auto-ubiquitinated Rsp5 *in vitro.* Moreover, similar to its mammalian homologues such as Nedd4-2, binding to a substrate or the Rup1 cofactor markedly stimulates Rsp5 auto-ubiquitination activity *in vitro*, implicating cofactor binding and intra-molecular unfolding in the Rsp5 activation cycle. These data indicate that Ubp2 and Rup1 enable a previously unappreciated regulatory feedback mechanism in Rsp5, and suggest that a tightly-coordinated pattern of auto-ubiquitination and deubiquitination forms a universal mechanism in the regulation of E3 ligase activity regardless of effects on enzyme stability.

## Results

### Rsp5 is Ubiquitinated and Deubiquitinated by Ubp2 *in vivo*

As previous studies [1], [2] had shown that Ubp2 interacts both physically and functionally with Rsp5, we examined a possible role of Ubp2 in modulating the ubiquitination status of Rsp5. We hypothesized that Ub-Rsp5 conjugates are transient and hence might only accumulate in DUB deficient cells. To assess this, we looked for evidence of ubiquitination by Western blotting of N-terminally HA-tagged Rsp5, expressed under control of its endogenous promoter [36], in soluble whole cell extracts prepared from both wildtype and an otherwise isogenic *ubp2* null mutant haploid yeast strains. The cell-free lysates were prepared in the presence of DUB and general protease inhibitors, and HA-Rsp5 was immunoprecipitated in native conditions prior to SDS-PAGE to enhance detection sensitivity.

**Figure 1:**
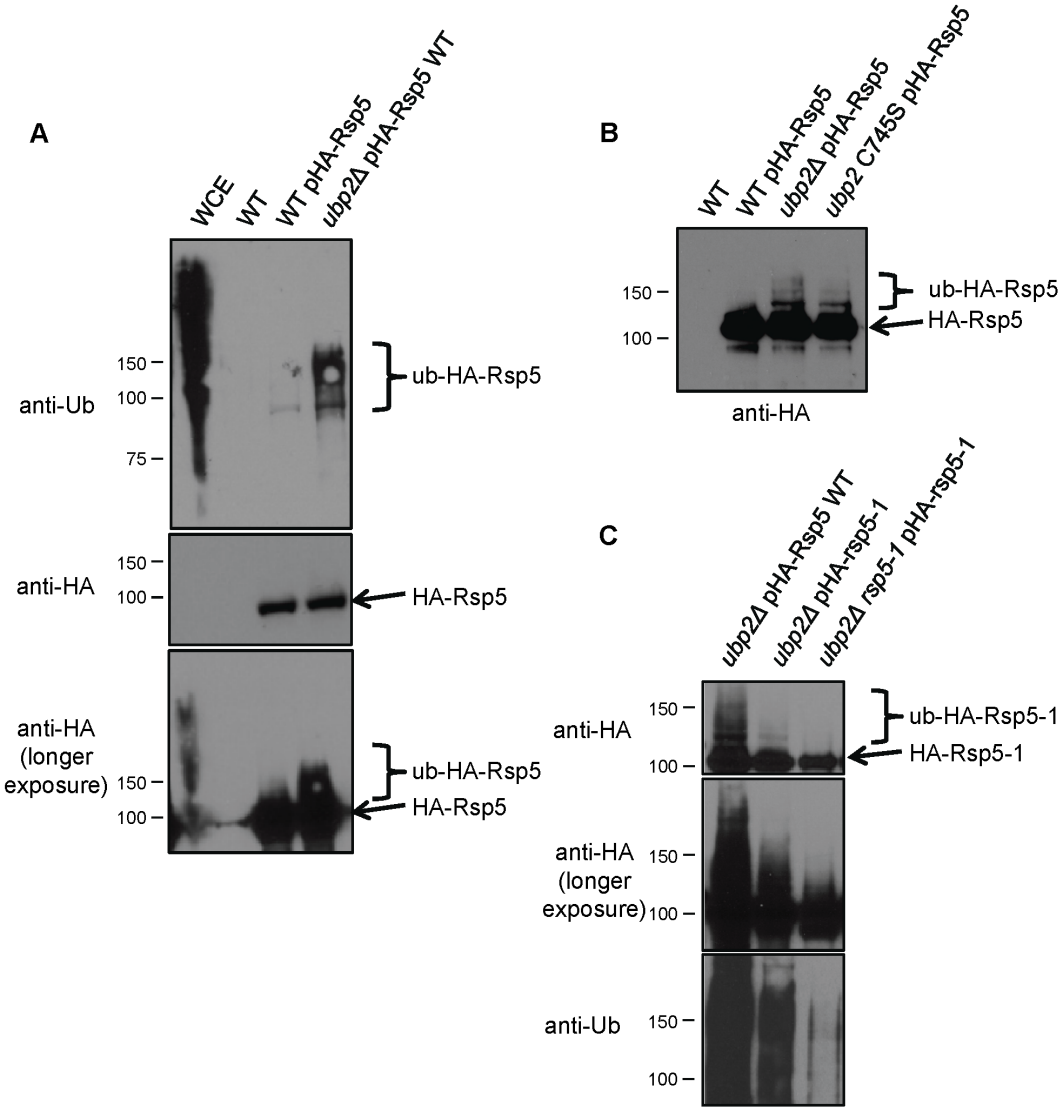
Rsp5 is stably ubiquitinated in the absence of Ubp2. (A) Western blot showing ubiquitinated and unmodified forms of Rsp5 *in vivo*. A plasmid expressing epitope-tagged Rsp5 (pHA-Rsp5) was transformed into wildtype and *ubp2*Δ cells. Cells were lysed in native buffer, and Rsp5 immunoprecipitated with anti-HA antibodies. After transfer to nitrocellulose, Rsp5 species were visualized using anti-ubiquitin (top panel) and anti-HA antibodies (middle; longer exposure at bottom). Whole cell extract from cells lacking plasmid (WCE) and immunoprecipitates from these same cells (WT) were run as positive and negative controls, respectively. (B) Experiment performed as in (A), except with an increased exposure time in order to visualize lower mobility Ub-HA-Rsp5 species. Accumulation of ubiquitinated HA-Rsp5 is evident in a strain bearing a catalytically inactive mutant allele in *UBP2* (*ubp2 C745S*). (C) Rsp5 is auto-ubiquitinated. Plasmids expressing either wildtype Rsp5 (pHA-Rsp5 WT) or a conditional catalytic mutant of Rsp5 (pHA-rsp5-1) were transformed into haploid yeast lacking *UBP2* alone or both *UBP2* and a fully functional copy of *RSP5* (*rsp5-1*). Prior to analysis, the strains were grown overnight, diluted in fresh media, and incubated at the non-permissive temperature (37°C) to inactivate Rsp5. Cell lysates were immunoprecipitated with anti-HA antibodies, and the blot first probed with anti-ubiquitin, followed by extended wash steps to rinse off residual antibody, and finally Rsp5 was detected with anti-HA antibodies. A darker exposure of the anti-HA blot is shown to highlight the lack of low-mobility (multi/poly-ubiquitinated) forms of Rsp5-1.

Whereas only a single dominant molecular weight species was apparent in WT cell derived extracts (Fig. 1A, centre and bottom panels), anti-ubiquitin immunoblotting revealed the presence of lower-mobility Rsp5 protein forms corresponding in size to mono and poly/multi-ubiquitinated in extracts isolated from *ubp2*Δ cells (Fig. 1A, top panel). These lower-mobility forms are not simply ubiquitinated proteins binding to Rsp5, as they were also detected with anti-HA antibody (Fig. 1A, bottom panel). Although only a small fraction of Rsp5 was ubiquitinated, these results nevertheless suggest that Rsp5 is indeed normally ubiquitinated transiently *in vivo* and that Ubp2 serves to deubiquitinate Rsp5.

### The Catalytic Activity of Ubp2 is Required for the Deubiquitination of Rsp5

One interpretation for the above data is that Ubp2 may be responsible for the removal of Ub chains from Rsp5. To test this, we examined whether the catalytic function of Ubp2 was required for this effect by monitoring the ubiquitination status of Rsp5 in a strain bearing a cysteine-to-serine point mutation at residue 745 of the genomic copy of Ubp2, a critical residue of the core conserved Cys box motif essential for catalytic activity [37]. Indeed, mobility shifted Ub-Rsp5 species accumulated in the *ubp2-C745S* mutant strain to a level equivalent to that of an *ubp2* deletion strain (Fig. 1B), confirming that Ubp2 enzyme activity is required for the removal of Ub from Rsp5.

### Rsp5 is Auto-ubiquitinated

Although the mammalian homologues of yeast E3 ligases have generally been reported to be auto-ubiquitinated [24], [38], examples of cross-ubiquitination by other E3 ligases have also been documented [39]. Hence, to determine if the ubiquitin modification on Rsp5 is the result of auto-ubiquitination or of the activity of another (unknown) E3 ligase, we examined the effects of inactivation of Rsp5 catalytic activity on the formation of ubiquitination species. To this end, we transformed a plasmid bearing an HA-tagged version of the conditional hypomorphic mutant *rsp5-1* allele (HA-*rsp5-1*) into yeast mutants deleted for *UBP2*. HA-tagged Rsp5-1 protein was then immunoprecipitated using anti-HA antibody and its ubiquitination status examined by Western blotting. Strikingly, cells expressing the Rsp5-1 mutant variant showed a drastic reduction in the level of mobility-shifted Ub-Rsp5 species compared with cells expressing wildtype HA-tagged Rsp5 (Fig. 1C). This result implies that, like most mammalian E3 ligases examined, Rsp5 is auto-ubiquitinated, possibly via an intra-molecular (*cis*) reaction.

We reasoned that the low level of ubiquitination still detectable likely stems from residual *rsp5-1* enzyme activity. However, since Rsp5 has the ability to interact with itself (i.e. dimerize) [5], it could also reflect modification in *trans* via dimerization with endogenous Rsp5 present in these same cells, or possibly by an alternate ligase. To address this, we expressed and purified HA-Rsp5-1 from a yeast mutant strain wherein the native *RSP5* locus had likewise been converted to a conditional allele (*ubp2*Δ *rsp5-1*). In this context, ubiquitinated Rsp5 was virtually undetectable when the double mutant was grown at a non-permissive temperature, which was confirmed by the lack of cross-reactivity with anti-ubiquitin antibody, unlike the previous two lanes (Fig. 1C). Therefore, the minor residual ubiquitination seen in *ubp2*Δ cells is most readily explained as an inter-molecular (i.e. *trans*) ubiquitination reaction by endogenous (i.e. wildtype) Rsp5.

Taken together, these data collectively indicate that the Rsp5 ubiquitinated forms detected *in vivo* are likely not due to the activity of another E3, and that Ubp2 inactivation is essential to reveal the innate intra-molecular (*cis*) ubiquitination process occurring on Rsp5.

### Substrate Recognition Stimulates Rsp5 Auto-ubiquitination

In the case of the mammalian HECT E3 Nedd4-2, the ligase is thought to remain in a folded, catalytically inactive conformation in the absence of a substrate, presumably through intra- or inter-molecular binding between the WW domain(s) and a PY motif in the HECT domain [21]. However, once a substrate binds to Nedd4-2, this inhibitory interaction is proposed to become disrupted, causing a change in the enzyme leading to an active conformation [21].

**Figure 2:**
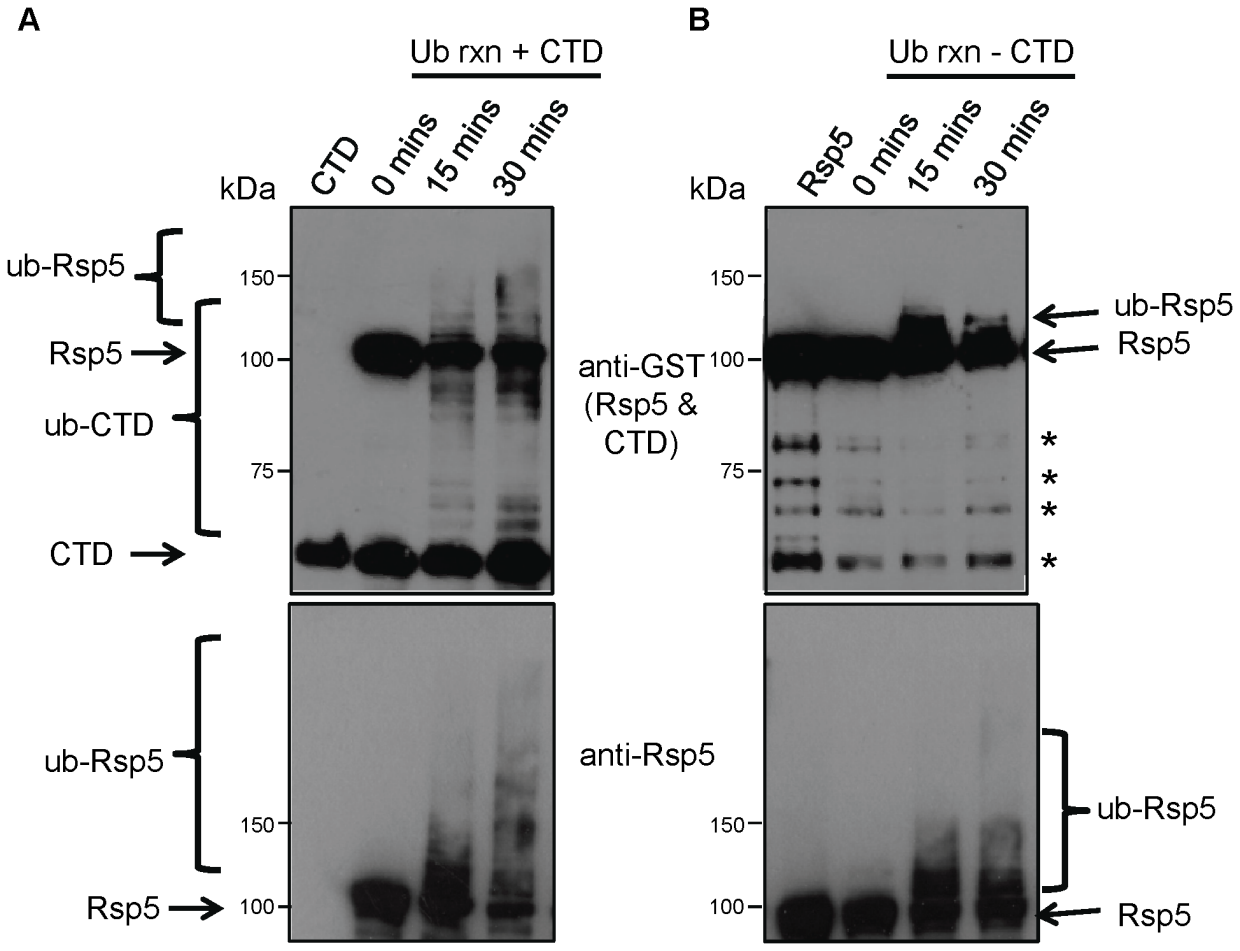
Substrate-induced Rsp5 auto-ubiquitination. Western blot analysis of an *in vitro* Rsp5 ubiquitination reaction in the (A) presence (+CTD lanes) or (B) absence of substrate (-CTD lanes). Reactions (E1, E2, Rsp5, ATP, buffer, Ub,+/− CTD) were incubated from 15 to 30 minutes as indicated, and stopped by the addition of sample loading buffer. Both GST-Rsp5 and GST-CTD were detected with anti-GST antibody, and Rsp5 (unmodified and modified forms) detected on a separately run blot with anti-Rsp5 antibody. Asterisks in (B, upper panel) denote degradation products of Rsp5. These bands are not present in (A, upper panel), as they are obscured by the CTD band. Ubiquitinated and unmodified forms of Rsp5 and CTD are indicated.

To explore whether this mechanism is likewise conserved in Rsp5, we performed an *in vitro* auto-ubiquitination reaction both in the absence and presence of substrate. For the reaction, we combined recombinant full-length Rsp5, E1 and E2 enzymes purified from *E. coli*, and ubiquitin, with or without a model substrate, the carboxy-terminal domain of RNA polymerase II (CTD) also purified from *E. coli* [40] (Fig. 2). RNAPII is ubiquitinated by Rsp5 *in vivo* in response to transcriptional arrest [41], [42], and the unphosphorylated form has been reported to be an excellent substrate *in vitro* [43]. Ubiquitination was initiated by the addition of ATP and allowed to proceed at room temperature for various periods of time to monitor progression. The ubiquitination of both Rsp5 (GST-Rsp5) and the substrate (GST-CTD) were monitored by Western blotting using antibodies directed against the GST tag and Rsp5 itself.

As expected, ubiquitinated CTD appeared upon addition of ATP, with higher molecular weight species indicating multi or poly-ubiquitination increasing proportional to reaction time (Fig. 2A, top panel). Auto-ubiquitination of Rsp5 also increased over time, with the non-modified form correspondingly decreasing in abundance. Strikingly, the substrate containing reactions (Fig. 2A, bottom panel) resulted in a markedly enhanced amount of shifted GST-Rsp5, corresponding to both mono-ubiquitinated and more extensively auto-ubiquitinated forms, as compared to the lanes in which no CTD was added (Fig. 2B, bottom panel). By 30 minutes, there is a notable accumulation of low-mobility forms, likely corresponding to Rsp5 conjugated to multiple ubiquitins representing either multi-ubiquitination or long poly-ubiquitin chains. In contrast, in the absence of substrate, only less extensively modified Rsp5 species were detectable, potentially corresponding to mono and short ubiquitin chains. Together, these results suggest that the presence of a substrate increases Rsp5 ubiquitination activity, consistent with a possible conformation change analogous to that reported for mammalian Nedd4-2.

### The Ubp2 Cofactor Rup1 Stimulates Rsp5 Auto-ubiquitination

**Figure 3:**
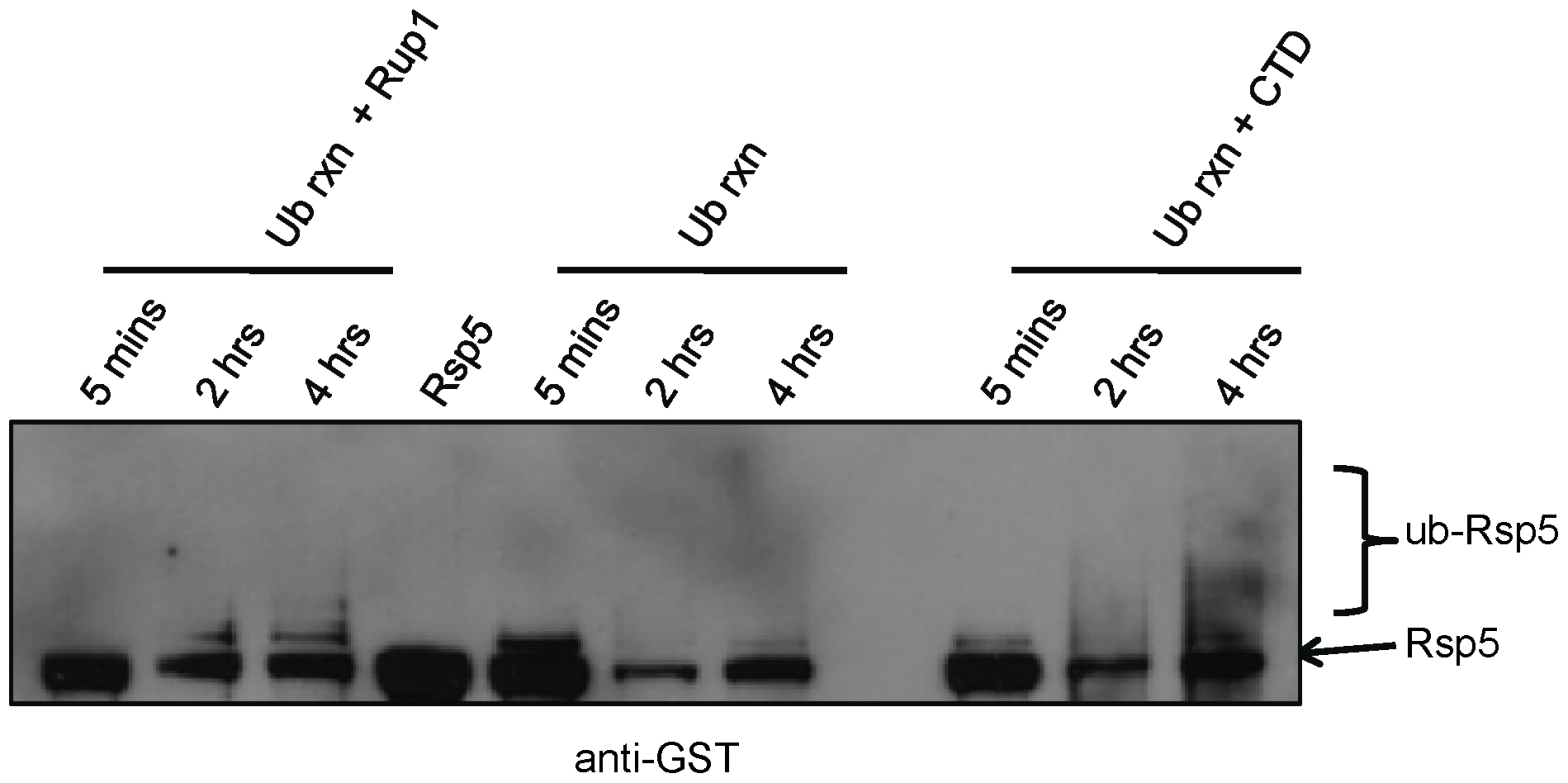
Rup1 stimulates Rsp5 auto-ubiquitination. Western blot analysis of an *in vitro* Rsp5 ubiquitination reaction with CTD (+CTD lanes) or lacking CTD in the presence of Rup1-TAP (+Rup1 lanes) purified from a strain lacking *UBP2*. Reactions were incubated from 5 minutes to 4 hours as indicated and stopped by the addition of sample loading buffer. Ubiquitinated and unmodified forms of GST-tagged Rsp5 were detected with anti-GST antibody.

Since we and others have shown that Rup1 binds to Rsp5 directly [1], [2], we next examined if Rup1 exerts an analogous influence on the auto-ubiquitination of Rsp5 *in vitro*. To this end, Rup1 was affinity-purified from a TAP-tagged yeast strain lacking a functional copy of *UBP2* to remove the possible confounding effects of deubiquitination. Even in the absence of substrate, we found that addition of a sub-stoichiometric amount of Rup1-TAP to the Rsp5 *in vitro* ubiquitination mixture described above resulted in a significant increase in the rate of accumulation of band intensities corresponding to mono and multi/poly-Ub-Rsp5 species as compared to reactions in which Rup-TAP was omitted (Fig. 3). The effect of Rup1 on Rsp5 auto-ubiquitination was qualitatively similar, albeit less pronounced, to that seen following addition of a molar excess of CTD substrate.

Taken together, these data show that, analogous to its mammalian homologue Nedd4-2 [21], Rsp5 auto-ubiquitination is stimulated both by the presence of substrates or cofactors. Hence, upon binding, Rsp5 may undergo an induced conformational change, causing an increase in its catalytic activity that leads in turn to both enhanced autocatalysis and substrate-directed ubiquitination. While future studies are needed to confirm this model, these results suggest that Rup1 exerts a regulatory effect independent of simply facilitating the recruitment or tethering of Ubp2 to Rsp5.

### Ubp2 Deubiquitinates Auto-ubiquitinated Rsp5 *in Vitro*

Our *in vivo* data (Fig. 1) indicate that auto-ubiquitinated Rsp5 is stabilized in the absence of Ubp2. This suggests that Ubp2 deubiquitinates Rsp5, thereby minimizing the accumulation of auto-ubiquitinated species. To test this directly, we examined the ability of purified TAP-tagged Ubp2 (Ubp2-TAP) isolated from an otherwise wild-type yeast strain (i.e. together in a complex with endogenous Rup1) to deubiquitinate Ub-GST-Rsp5 species *in vitro*. First, we assayed for deubiquitination activity *in vitro* by incubating various amounts of the purified Ubp2 preparation with K63-linked poly-ubiquitin chains, which Ubp2 has previously been reported to cleave [44]. As predicted, Ubp2 was able to deubiquitinate poly-Ub chains down to mono-ubiquitin, and could be fully blocked by the addition of the DUB inhibitor ubiquitin aldehyde (Fig. S1).

Next, we examined the ability of Ubp2 to deubiquitinate Ub-Rsp5 generated in an *in vitro* reaction in which substrate (CTD) was added to stimulate auto-ubiquitination (evident as an extensive smear by Western blot). Affinity-purified Ubp2-TAP, alone or with a DUB inhibitor (ubiquitin aldehyde) included as a control, or buffer alone, were then added, and the mixture incubated for an additional 2 hours. The ubiquitination status of Rsp5 was then monitored by Western blotting.

**Figure 4:**
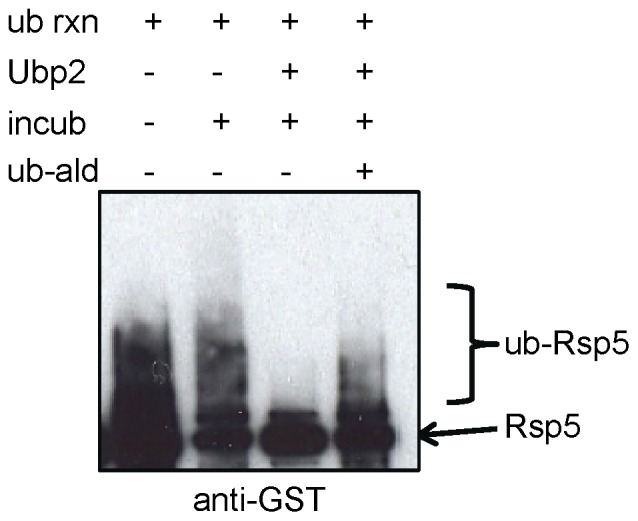
Ubp2 deubiquitinates Rsp5 *in vitro.* Western blot showing Ubp2 reversal of mobility shifted Rsp5-Ub species. Ubiquitination reactions were performed in the presence of substrate (CTD) to stimulate Rsp5 auto-ubiquitination (+ub rxn). Ubp2-TAP (+Ubp2), buffer only (-Ubp2), and/or the DUB inhibitor ubiquitin aldehyde (+ub-ald) were then added, and the reactions allowed to incubate for a further 2 hours (+incub). Ubiquitinated and unmodified forms of GST-tagged Rsp5 were detected with anti-GST antibody.

As seen in Figure 4, the addition of Ubp2-TAP led to a dramatic reduction in the level of auto-ubiquitinated Rsp5, and a corresponding increase in both non-ubiquitinated and mono-ubiquitinated Rsp5 compared to control (i.e. buffer or inhibitor). These results establish Ubp2 as a direct regulator of Rsp5. While the Ubp2 preparation was, however, less effective at removing residual short ubiquitin chains and the terminal ubiquitin on Rsp5, our observations are in line with a previously reported preference of Ubp2 for processing longer ubiquitin chains [44].

## Discussion

### Detection of a Novel Ubiquitin Modification on Rsp5

It would appear that while most E3 ubiquitin ligases are auto-ubiquitinated, they also associate with deubiquitinating enzymes. Stably bound DUBs appear to reverse auto-ubiquitination [33], [34], although they may also deubiquitinate E3 substrates as well [45], [46]. In this study, we show that, similar to its mammalian homologues, yeast Rsp5 is transiently auto-ubiquitinated. Although this modification has previously been detected, our work provides the first in-depth analysis of Rsp5 auto-ubiquitination *in vivo*, implying the modification may be biologically significant. Rsp5 auto-ubiquitination may have been difficult to detect previously in a physiological context because it is rapidly reversed by Ubp2. We found that a catalytically inactive Ubp2 point mutant, *ubp2C745S,* accumulates Ub-Rsp5 comparable to a full *ubp2* deletion. Moreover, affinity purified Ubp2 is able to deubiquitinate auto-ubiquitinated Rsp5 *in vitro*. Although a significant portion of Rsp5 remains mono-ubiquitinated, this is consistent with the previously identified catalytic activity of Ubp2 which has a preference for longer ubiquitin chains [44]. Previous studies have shown differences in the function of a protein that was mono- versus poly-ubiquitinated (ex. RNA polymerase II ubiquitination [47] and PCNA ubiquitination as reviewed in [48]). Therefore, the removal of ubiquitin by Ubp2, even if not complete, still has the potential to modify Rsp5 function. In addition, it is also quite possible that a larger subset of cellular Rsp5 may be ubiquitinated *in vivo* that was not visible under the conditions of our experiments. For example, this could occur if ubiquitination is transient or if deubiquitinating enzymes other than Ubp2 (of which there are 15 other candidate Ubps in yeast) are likewise responsible for cleavage of ubiquitin from Rsp5 under certain circumstances.

It is currently unknown whether Rsp5 is subject to long chain (poly-ubiquitination), multi-site ubiquitination, or if several short linked chains are attached. Since most blots of ubiquitinated Rsp5 show several discrete bands, it seems likely that poly- or multi-ubiquitination occur. It may be possible to determine the type of ubiquitin chain present on Rsp5, either by using chain type specific antibodies [49], [50], tandem mass spectrometry, or a panel of site–specific lysine mutant ubiquitin chains to prime the *in vitro* ubiquitination reaction. Relevant to this, Rsp5 and Ubp2 have been reported to catalyze the ubiquitination and deubiquitination [44], respectively, of K63-linked chains, which suggests the presence of auto-ubiquitinated K63-linked chains on Rsp5.

### Rsp5 is Auto-ubiquitinated through an Intra-molecular Mechanism

Another avenue to consider is the molecular mechanism by which the auto-ubiquitination reaction occurs. From previous studies on mammalian ubiquitin ligases [24], [34], [38], [51], the attachment of a ubiquitin chain to the ligase can either result from an intra-molecular reaction, in that the activated enzyme itself transfers ubiquitin to a residue on the same molecule, or as an inter-molecular reaction, wherein the ligase will ubiquitinate another enzyme molecule, such as another subunit of a dimer. From our results presented in this study, it seems that ubiquitination on Rsp5 happens preferentially as an intra-molecular reaction *in vivo*, although some low level of *trans* ubiquitination does occur as the remaining (a small percentage of the total) Ub-Rsp5-1 smear is eliminated when the genomic wildtype copy of Rsp5 is deleted. *Trans* ubiquitination of Rsp5 is, however, consistent with reports showing that Rsp5 has the ability to form dimers [5]. The disappearance of detectable Ub-Rsp5-1 species in the absence of functional endogenous Rsp5 also implies that no other ubiquitin ligases can stably ubiquitinate Rsp5 under standard yeast culture growth conditions, at least with a high enough efficiency to generate a band detectable by Western blot analysis.

### Binding to Substrates or Cofactors may Unfold and Activate Rsp5

Given that intra-molecular ubiquitin transfer seems to be the most plausible route by which Rsp5 becomes auto-ubiquitinated, we wanted to determine if an Rsp5 substrate, such as a recombinant form of the carboxy-terminal domain (CTD) of RNA polymerase II, influences the rate or degree of auto-ubiquitination of Rsp5. As evidenced by our *in vitro* data, the presence of either CTD or the cofactor Rup1 resulted in increased Rsp5 auto-ubiquitination over time, with the Ub-Rsp5 abundance elevated in terms of chain length and/or the number of attached ubiquitins. This effect can be compared to reactions without ectopic substrate, for which only mono-Ub or a short poly-Ub chain was detected. The increase in more extensively ubiquitinated forms, which is especially striking in reactions containing CTD, may indicate either increased processivity by Rsp5 or an increased preference for the formation of polyubiquitin chains under this condition. It is important to note that Rup1 has been reported to act as a cofactor for Ubp2, increasing the *in vitro* deubiquitinating activity of Ubp2 [1]. It would be interesting to determine the overall impact of Rup1 on the Ubp2-Rup1-Rsp5 complex by examining the activity of Ubp2 on Rsp5 without Rup1 present.

Since the addition of an Rsp5 substrate markedly increases Rsp5 auto-ubiquitination, it is tempting to speculate that, similar to the model proposed for its mammalian homologue Nedd4-2 [21], that Rsp5 is maintained in a “non-active” conformation or a poised folded state in the absence of suitable substrates or co-factors such as Rup1. This “inhibited” condition, as in the case with Nedd4-2, could be due to an intra- or inter-molecular interaction between one or more WW domains in the middle of Rsp5 with a sequence in the HECT domain. As previously reported [40], Rsp5 possesses a putative ‘LPQY’ motif in the HECT domain, similar to mammalian Nedd4 family ligases, which may serve as a candidate for binding to WW domain-containing cofactors. Upon recruitment of a binding partner or a substrate such as RNAPII CTD to the enzyme, Rsp5 may become unfolded as the WW domain shifts to bind the new factor, allowing the HECT-located PY motif on Rsp5 to become displaced such that the catalytic activity is engaged and the HECT domain is free to ubiquitinate both the substrate and Rsp5 itself.

Evidence, from this study and others [25], has established that proteasomal degradation is an unlikely fate for Ub-Rsp5, as catalytic mutants of Rsp5 are as stable as the wildtype forms. Given that ubiquitinated Rsp5 is seemingly not rapidly degraded by the proteasome, if poly-ubiquitination occurs it likely involves K63 chains consistent with the known catalytic activity of Rsp5. Recent studies, for example, on the ubiquitination of the mammalian Itch, has shown that ligase ubiquitination results in an as yet unknown non-degradative fate for the enzyme [24]. The ubiquitination of Rsp5, then, may affect its function in a non-catalytic manner. For example, it could serve as a negative feedback mechanism, in which the presence of an Rsp5 substrate leads to the ubiquitination and inactivation of Rsp5 back to a resting ground state immediately after ubiquitination of the target.

On the other hand, physical association of Rsp5 with Ubp2 would result in the deubiquitination and re-poising of Rsp5. Ubiquitination of Rsp5 may result in a decrease in enzymatic activity, possibly through a conformation change in the ligase. Auto-ubiquitination of Rsp5 may proceed until a suitable Ub chain length is reached for the inhibition of Rsp5 activity. Specifically, since the deletion of *UBP2* somewhat paradoxically causes a phenotype of Fur4 internalization similar to a substrate level ubiquitination defect [2], the accumulation of ubiquitinated Rsp5 *in vivo* (albeit to substoichiometric levels) may result in a down-regulation of Rsp5 ligase activity at the MVB, leading to the protein trafficking defect observed in *ubp2* mutant cells. Along with the modification of Rsp5 activity, Ubp2, as recently reported [8], is also involved in the deubiquitination of ESCRT proteins. Ubp2-dependent deubiquitination was found to be required for proper MVB sorting [8]. The sum of these two roles of Ubp2 may result in the ultimate fate of an endocytosed protein at the MVB.

Apart from a possible role in the direct modification of Rsp5 activity, it is also possible that auto-ubiquitination could result in the subcellular re-localization of Rsp5 protein to another cell compartment. Rsp5 has been reported to be present in various parts of the cell, most notably along the endocytic and vesicle trafficking pathways [52]. Recent studies have implicated ubiquitination as an important signal specifying the intracellular localization of proteins [53]. Therefore, it is possible that auto-ubiquitination of Rsp5 could represent one signal by which Rsp5 is re-directed to various compartments and new roles after ubiquitinating a substrate. “Activation” of Rsp5 by Ubp2 deubiquitination in this case then, may actually occur through a change in the localization of Rsp5, and therefore a change in possible Rsp5 substrates.

Another possibility is that auto-ubiquitination prevents binding of Rsp5 to one or more of its co-factors, interacting proteins, or even certain substrates. In this case, auto-ubiquitination would lead to differential substrate recognition or result in a change in Rsp5 activity. This could be tested easily by monitoring the binding of various known co-factors and substrates of Rsp5 to both ubiquitinated and non-ubiquitinated forms of Rsp5. In this case, the ubiquitination of Rsp5, and the reverse, deubiquitination by Ubp2, could therefore lead to shuttling between pathways or processes due to differential protein binding.

The exact ubiquitination site(s) on Rsp5 is currently unknown, although candidate lysines, specifically in the C2 and first WW domain, have been identified in at least one recent large scale study [26]. Ubiquitination at these sites, therefore, may result in the translocation of Rsp5 from one cell compartment to another, or affect binding of Rsp5 to WW domain-binding cofactors. Future mapping experiments will be required to determine the effect the modification(s) have on Rsp5 folding, localization, and protein-protein interactions. Whichever case it may be, modification of Rsp5 by ubiquitin seemingly only affects a subset of the overall Rsp5 present in a cell. It is possible that only this subset is accessible to cofactors or substrates, resulting in Rsp5 activation and subsequent auto-ubiquitination, which, in turn, may lead to the fates discussed above.

Taken together, the data presented in this study suggest that deubiquitinating enzymes in yeast, similar to well characterized DUBs in mammalian systems, have complex and important roles in regulating ubiquitination through direct modification of a ubiquitin ligase.

## Materials and Methods

### Strains and Plasmids

A yeast expression plasmid encoding wild-type *RSP5* under control of its natural promoter pHA-Rsp5(p[HA-*RSP5*, *CEN*, *LEU2*]) [36] and the catalytic mutant clone pHA-rsp5-1(pPC33) were obtained from Teresa Zoladek. Bacterial expression plasmids for generating recombinant proteins pET15b-UBC4, pET21a-GST-TEV-CTD, pGEX-6P2-RSP5 are described in [40]. A strain carrying a *UBP2* allele (*ubp2-C745S*) in the BY4741 strain background, encoding catalytically inactive Ubp2, was constructed in two steps: First, we disrupted the endogenous wildtype *UBP2* locus with a targeting cassette encoding *URA3* in a haploid uracil-auxotroph, creating an *ubp2*::*URA3* deletion allele. Then, sequences surrounding the *ubp2C745S* mutation in the yeast expression plasmid pF/H426-*ubp2*-*C745S* [2] were PCR amplified and transformed into the *ubp2*::*URA3* strain. Integrants lacking a functional *URA3* marker were selected for on media containing 5-Fluoroorotic Acid (5-FOA). To eliminate possible secondary/unintended mutations, 5-FOA transformants were individually mated to haploid mat α *ubp2*Δ cells, diploids were selected, and haploid segregants scored after tetrad dissection. Clones carrying the *ubp2 C745S* allele showing a growth rate comparable to wildtype were identified by genomic amplification and sequencing of the modified *UBP2* locus. The TAP strains Rup1-TAP *ubp2*Δ and Ubp2-TAP are as described in [2].

### Cell Lysis, Immunoprecipitation, and Western Blots

Yeast cells were cultured, harvested and lysed by bead beating essentially as previously described in [2] in the presence of 10 mM of the DUB inhibitor N-ethylmaleimide (NEM; Sigma) and 0.1 nM of the proteasome inhibitor MG132 (Sigma). For anti-HA immunoprecipitations, approximately 6 mg of soluble cell-free protein lysate was first pre-cleared for 2 hrs at 4°C with protein-G agarose (Millipore), followed by immunoprecipitation with mouse monoclonal anti-HA antibody (generated from a cultured 12CA5 cell line; a kind gift from Mike Tyers, Samuel Lunenfeld Research Institute, Mount Sinai Hospital, Toronto, Ontario) and 20 µl of a 50% slurry of protein-G beads (Millipore) for 1hr. After extensive bead washing with lysis buffer, bound proteins were eluted using SDS sample loading buffer, subjected to PAGE, and transferred onto a nitrocellulose membrane as described in [2]. Western blots were probed with either mouse monoclonal anti-GST antibody (B-14, Santa Cruz Biotechnology; 1∶2000 dilution), mouse monoclonal anti-ubiquitin antibody (clone 6C1; Sigma; 1∶5000 dilution), or rabbit anti-Rsp5 antiserum [54]. Incubation with secondary antibody and detection of target proteins by ECL were then performed.

### Purification of Recombinant Ubc4, Rsp5, and CTD

Inducible bacterial expression vectors bearing HIS-*UBC4*, GST-*CTD*, and GST-*RSP5* fusion cassettes were transformed into *E. coli* strain BL21(DE3) and recombinant proteins were expressed and affinity purified essentially as described in [40] with some modifications. Cells were grown in LB media at 37°C with vigorous shaking to an OD_590_ of 0.6, and 1 mM isopropyl-β-1-thio-D-galactopyranoside (IPTG) added to induce protein expression. Cells were incubated for a further 12 hrs at 16°C, then pelleted and lysed in sonication/binding buffer (20 mM HEPES, pH 8.0, 500 mM NaCl, 10% glycerol, and 0.5 mM tris(2-chloroethyl) phosphate (TCEP)) containing a protease inhibitor cocktail (Complete, Roche). For HIS-*UBC4*, 5 mM imidazole was added to the buffer to minimize non-specific binding during purification.

After sonication, whole cell lysates were clarified by centrifugation at 80,000×g for 1 h at 4°C, and soluble protein incubated with either nickel-nitrilotriacetic acid agarose (Ni-NTA, Qiagen) to isolate the HIS-UBC4 fusion protein, or glutathione sepharose (Amersham) to recover the GST-tagged proteins. Beads were washed repeatedly with sonication/binding buffer (with 30 mM imidazole added to minimize non-specific background for the Ni-NTA purification). Bound HIS-*UBC4* was eluted with sonication/binding buffer containing 500 mM imidazole, while the GST-tagged proteins were eluted with sonication/binding buffer containing 15 mM glutathione. Protein eluates were concentrated to around 1 mg/ml by centrifugal filtration (MW cutoff 10 kDa; Microcon, Amicon-Millipore) as needed, and the purity and yield assessed by electrophoresis together with BSA control protein to estimate concentration on a 4–12% Bis-Tris polyacrylamide gel (Invitrogen) followed by Coomassie staining (Fig. S2).

### TAP Tag Purifications and Deubiquitinating Activity Assay

Tandem affinity purification of endogenous Ubp2-TAP and Rup1-TAP fusion proteins were performed on 4L yeast cultures essentially as described in [55], except the final elution buffer was DUB reaction buffer (10% glycerol, 50 mM Tris-Cl pH 7.9, 50 mM NaCl, 1 mM EDTA, 2 mM DTT, and 3 mM EGTA). Yield and purity of the purifications were estimated by SDS-PAGE followed by staining with silver. To confirm the deubiquitination activity of the Ubp2-TAP preparation, 1–30 µl of the TAP preparation (0.5 µg/ml) was incubated with 1.5 µl K63-linked ubiquitin chain (Boston Biochem) (2 mg/ml). The reaction was incubated for 1 hour at room temperature to allow for deubiquitination, and stopped by the addition of 3.6 µl of 100% trichloroacetic acid (TCA), followed by incubation on ice for 30 mins, and centrifugation to collect precipitated protein. Precipitates were resuspended in sample loading buffer, along with unbuffered Tris to neutralize the sample. The above was repeated but with 1–5 µl of ubiquitin aldehyde (1 mg/ml) to confirm that deubiquitination was Ubp dependent.

### *In vitro* Ubiquitination and Deubiquitination Assays

Ubiquitination assays in microtube format were done essentially as described in [40] with the following modifications. Each reaction (15 µl total volume) consisted of 5x assay buffer (250 mM HEPES, pH 7.4, 25 mM MgOAc, 2.5 mM TCEP, 500 mM NaCl, and 50% glycerol), 10 µg of purified yeast recombinant ubiquitin (Boston Biochem), 0.16 µg of recombinant commercial yeast E1 (Ube1; Boston Biochem), 3.8 µg of purified E2 (HIS-Ubc4), 1.2 µg of purified E3 (GST-Rsp5), 8 pmol of purified substrate GST-CTD, and 3.3 mM ATP (Sigma). In reactions supplemented with tandem affinity-purified yeast Rup1, 3.9 µl of a dilute Rup1-TAP preparation was added. Water was added to each reaction to bring the final volume of all reactions to 15 µl. Finally, ATP was added last to initiate enzyme activity, at time = 0, to all reactions rapidly in succession. Samples were incubated at room temperature and stopped at indicated times by boiling with SDS-PAGE sample buffer.

For the deubiquitination experiments, samples were first subject to the ubiquitination conditions described above, except using 1 µg ubiquitin, and approximately 0.6 µg of a concentrated preparation of tandem-affinity purified yeast Ubp2-TAP, alone or together with 1 µg of the DUB inhibitor Ub-aldehyde (Affinity Research) as a negative control where indicated. Calmodulin elution buffer [55] was added to bring the final reaction volume up to a total of 30 µl. Reactions were then incubated for a further 2 hrs at room temperature to allow for deubiquitination to proceed, then stopped by boiling in sample loading buffer. The reaction products were analyzed by separation on a 4–12% SDS-PAGE gel and visualized by Western blotting using anti-GST antibody.

## Supporting Information

Figure S1 The Ubp2-TAP preparation is catalytically active. (A) K63-linked ubiquitin chain was incubated *in vitro* with varying amounts of TAP purified Ubp2 to check for deubiquitination activity. The reaction was stopped and proteins precipitated by the addition of trichloroacetic acid (TCA), electrophoresed and visualized by staining with silver. The grey arrow bar indicates decreasing amounts of Ubp2 added, and the grey bar indicates an equivalent amount of Ub chain added in each reaction. Arrows point to the location of the various mono and polyubiquitin species. Mono ubiquitin (Ub) was located at the dye front. (B) To test for DUB specificity, the experiment was repeated, with an equivalent amount of Ubp2 and K63 chain in each reaction. Increasing amounts of ubiquitin aldehyde, a DUB inhibitor, was added to each reaction (grey arrow bar). The mobility of reference molecular weight markers is shown at the left. Mono and di-ubiquitin (Ub, Ub-Ub) were located at the dye front.
(TIF)

Figure S2 Purification of recombinant Ubc4, Rsp5, and CTD. HIS-Ubc4, GST-Rsp5, and GST-CTD were purified as described in materials and methods. The indicated volumes of the purified proteins, and indicated amounts of bovine serum albumin (BSA) standards were electrophoresed on a SDS-PAGE gel, followed by Coomassie staining to visualize proteins. Protein concentrations of the purified preps were estimated by comparisons with the BSA standard.
(TIF)
