# Supplementary figures and images for: Ubp2 Regulates Rsp5 Ubiquitination Activity In Vivo and In Vitro
Source: PLoS One. 2013 Sep 19;8(9):e75372. doi: 10.1371/journal.pone.0075372 (PMC3777918; doi:10.1371/journal.pone.0075372)

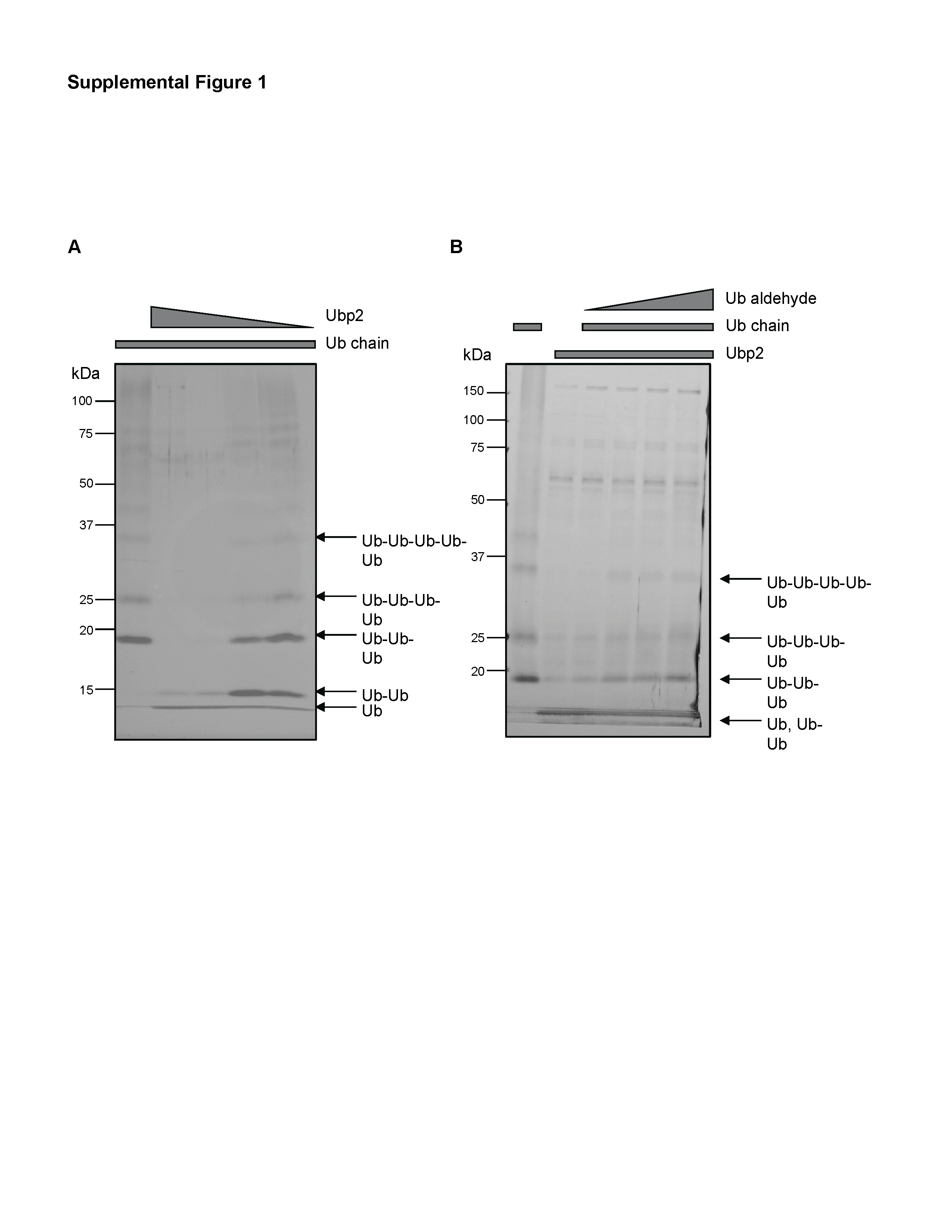

Supplement: Figure S1 — The Ubp2-TAP preparation is catalytically active. (A) K63-linked ubiquitin chain was incubated in vitro with varying amounts of TAP purified Ubp2 to check for deubiquitination activity. The reaction was stopped and proteins precipitated by the addition of trichloroacetic acid (TCA), electrophoresed and visualized by staining with silver. The grey arrow bar indicates decreasing amounts of Ubp2 added, and the grey bar indicates an equivalent amount of Ub chain added in each reaction. Arrows point to the location of the various mono and polyubiquitin species. Mono ubiquitin (Ub) was located at the dye front. (B) To test for DUB specificity, the experiment was repeated, with an equivalent amount of Ubp2 and K63 chain in each reaction. Increasing amounts of ubiquitin aldehyde, a DUB inhibitor, was added to each reaction (grey arrow bar). The mobility of reference molecular weight markers is shown at the left. Mono and di-ubiquitin (Ub, Ub-Ub) were located at the dye front. (TIF) [file pone.0075372.s001.tif]

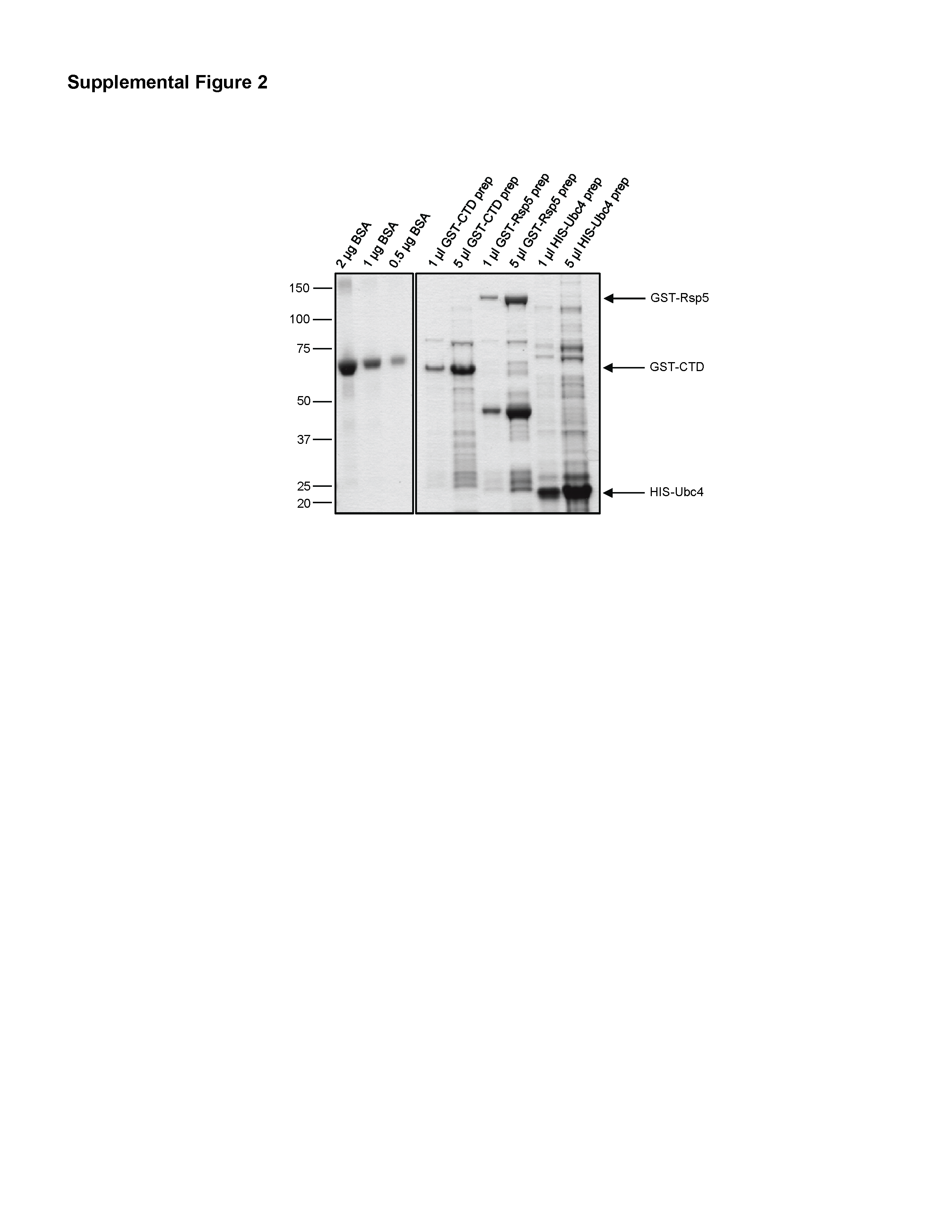

Supplement: Figure S2 — Purification of recombinant Ubc4, Rsp5, and CTD. HIS-Ubc4, GST-Rsp5, and GST-CTD were purified as described in materials and methods. The indicated volumes of the purified proteins, and indicated amounts of bovine serum albumin (BSA) standards were electrophoresed on a SDS-PAGE gel, followed by Coomassie staining to visualize proteins. Protein concentrations of the purified preps were estimated by comparisons with the BSA standard. (TIF) [file pone.0075372.s002.tif]
